# Extragingival pyogenic granuloma: a case report

**DOI:** 10.1186/1757-1626-1-371

**Published:** 2008-12-03

**Authors:** Maryam Amirchaghmaghi, Farnaz Falaki, Nooshin Mohtasham, Pegah Mosannen Mozafari

**Affiliations:** 1 Department of Oral Medicine, dental research center, dental faculty of Mashhad University of Medical Sciences, Mashhad, Iran; 2 Department of Oral Pathology, dental research center, dental faculty of Mashhad University of Medical Sciences, Mashhad, Iran

## Abstract

The pyogenic granuloma is thought to represent an exuberant tissue response to local irritation or trauma.

Clinically these lesions usually present as single nodule or sessile papule with smooth or lobulated surface. These may be seen in any size from a few millimeters to several centimeters. Pyogenic granuloma of the oral cavity is known to involve the gingiva commonly (75% of all cases). Rarely it may present extragingivally. Here, we report a case of Pyogenic granuloma in the palate of a 16 years old man which is very rare location for this lesion.

## Background

Pyogenic granuloma is a relatively common, soft tissue tumor of oral cavity that is belived to be reactive and not neoplastic in nature [1,2] the name pyogenic granuloma is a misnomer since the condition is not associated with pus and does not represent a granulma histologically [3-5] some authors use the term lobular capillary hemangioma for this lesion [6,7].

The pyogenic granuloma is thought to represent an exuberant tissue response to local irritation or trauma [2,4,5,8]

Clinically these lesions usually present as single nodule or sessile papule with smooth or lobulated surface. [1-3,5-7] These may be seen in any size from a few millimeters to several centimeters. [2,3,5-7,9] As lesions mature, the vascularity decreases and the clinical appearance is more collagenous and pink. [3] The peak prevalence is in teenagers and young adults, with a female predilection of 2:1 [1,3,5,10]

The increased incidence of these lesions during pregnancy may be related to the increasing levels of estrogen & progesterone. [1-3] Pyogenic granuloma of the oral cavity is known to involve the gingiva commonly (75% of all cases). Uncommonly it can occur on the lips, tongue, buccal mucosa, palate and so on. [2,4,5] The purpose of this article is to report an unusual case of extragingival pyogenic granuloma occurring on the hard palate.

## Case report

A 16 years old male patient was referred to our department with a chief complaint of a lesion on his hard palate.

The lesion was of negligible size when the patient first noticed it (3 months ago), but had grown rapidly over the past 20 days to attain the present size.

The patient's medical history was unremarkable. Clinical examination revealed an exophytic, pedunculated lesion that measured 0.7 cm in diameter, and in it's surface pseudomembraneuse with some areas of erythema was seen.

**Figure 1:**
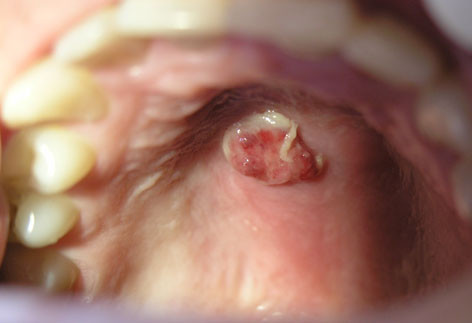
Clinical appearance: an exophytic pedunculated lesion with pseudomembrane on the surface.

**Figure 2:**
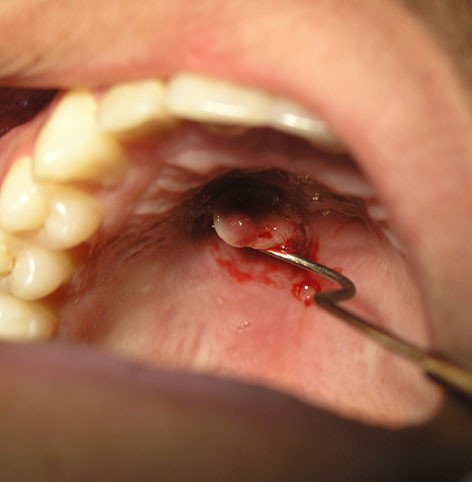
Clinical appearance: an exophytic pedunculated lesion with pseudomembrane on the surface.

The lesion was firm in consistency and non tender (fig 1), with minimal bleeding (fig 2). In addition, the patient had poor oral hygiene.

Due to the relatively small size of the lesion, an excisional biopsy, along with histopathologic evaluation was recommended as the diagnostic approach.

**Figure 3:**
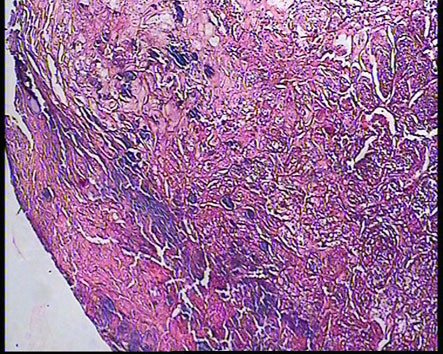
excisional biopsy showing granulation tissue: chronic inflammatory cell, blood vessels and collagen fibers.

The histopathologic examination revealed granulation tissue with non neoplastic proliferation of endothelial cells with blood cells formation and infiltration of acute and chronic inflammatory cells in a few collagenous matrix (fig 3). Surface of the lesion was consistent with hyperplastic parakeratinized stratified squamous epithelium with areas of atrophy and ulcer and fibrinoleukocytic membrane.

These findings were consistent with a histopathological diagnosis of pyagenic granuloma.

## Discussion

In the oral cavity pyogenic granulomas show a striking predilection for the gingiva, with interdental papillae being the most common site in 70% of the cases. They are more common in the maxillary anterior area than any other area in the mouth. Gingival irritation and inflammation that result from poor oral hygiene, dental plaque and calculus or over-hanging restorations may be precipitating factors in many cases. [2,8] Pyogenic granulomas of head and neck are uncommonly seen extragingivaly in areas of frequent trauma such as the lower lip, tongue and palate. [2,4] In the present case, the constant trauma inflicted by nuts on the hard palate could have been the etiology behind the growth on the palate. Such atypical presentation, like the case in discussion can be rather confusing and can lead to erroneous diagnoses of other more serious lesions. These include amelanotic melanoma, basal metastatic carcinoma and squamous cell carcinoma, Kaposi 's sarcoma and hemangioma.

Although pyogenic granuloma an be diagnosed clinically with considerable accuracy, radiographic and histopathological investigations, aid in confirming the diagnosis and treatment. Radiographs are advised to rule out bony destruction suggestive of malignancy or to identify a foreign body.

All clinically suspected pyogenic granulomas must be biopsied to rule out more serious conditions as mentioned previously. The histopathological picture of the extra gingival pyogenic granuloma is quit similar to the ones occurring on the gingival. Microscopically, it consists of many dilated blood vessels in a loose edematous connective tissue stroma. There is typically a dense acute inflammatory infiltration but this may be scanty or absent. [2,5,9]

Treatment of pyogenic granuloma consists of conservative surgical excision which is usually curative. There is a relatively high rate of recurrence (about 15%) after simple excision. [3] Recurrences after surgery of extragingival pyogenic granuloma is however uncommon [4]

## Consent

Written informed consent was obtained from the patient for publication of this case report and accompanying images. A copy of the written consent is available for review by the Editor-in-Chief of this journal written informed consent was obtained from the patient for publication of this report

## Competing interests

The authors declare that they have no competing interests.

## Authors' contributions

MA and FF carried out the clinical study and reviewed the manuscript, followed the patient and collected the documents. NM carried out the histopathologic examinations. PMM assisted in clinical study and drafted the manuscript.
